# Response Surface Methodology for the Optimisation of Electrochemical Biosensors for Heavy Metals Detection

**DOI:** 10.3390/bios9010026

**Published:** 2019-02-13

**Authors:** Giuseppe Egidio De Benedetto, Sabrina Di Masi, Antonio Pennetta, Cosimino Malitesta

**Affiliations:** 1 Dipartimento di Beni Culturali, Università del Salento, Via D. Birago 64, 73100 Lecce, Italy; giuseppe.debenedetto@unisalento.it (G.E.D.B.); antonio.pennetta@unisalento.it (A.P.); 2 Dipartimento di Scienze e Tecnologie Biologiche ed Ambientali, Via per Monteroni 1, 73100 Lecce, Italy; cosimino.malitesta@unisalento.it

**Keywords:** biosensors, enzyme inhibition, metal ions, central composite design, response surface methodology

## Abstract

Herein, we report the application of a chemometric tool for the optimisation of electrochemical biosensor performances. The experimental design was performed based on the responses of an amperometric biosensor developed for metal ions detection using the flow injection analysis. The electrode preparation and the working conditions were selected as experimental parameters, and thus, were modelled by a response surface methodology (RSM). In particular, enzyme concentration, flow rates, and number of cycles were reported as continuous factors, while the sensitivities of the biosensor (S, µA·mM^−1^) towards metals, such as Bi^3+^ and Al^3+^ were collected as responses and optimised by a central composite design (CCD). Bi^3+^ and Al^3+^ inhibition on the Pt/PPD/GOx biosensor response is for the first time reported. The optimal enzyme concentration, scan cycles and flow rate were found to be 50 U·mL^−1^, 30 and, 0.3 mL·min^−1^, respectively. Descriptive/predictive performances are discussed: the sensitivities of the optimised biosensor agreed with the experimental design prediction. The responses under the optimised conditions were also tested towards Ni^2+^ and Ag^+^ ions. The multivariate approach used in this work allowed us to obtain a wide working range for the biosensor, coupled with a high reproducibility of the response (RSD = 0.72%).

## 1. Introduction

Heavy metal ions are pollutants that seriously affect the health of the ecosystem due to their non-biodegradability and enrichment in the environment and biological systems [1]. Nowadays, the determination of heavy-metal ions in water samples represents an important issue. Different analytical techniques are employed for qualitative and quantitative detection of heavy metal ions [2], including atomic absorption spectroscopy (AAS), atomic fluorescence spectroscopy (AFS) and inductively coupled plasma mass spectrometry (ICP-MS) [3]. The above techniques have several advantages, such as high sensitivity, selectivity and accuracy. Otherwise, they present some problems, such as the complexity, cost and long steps of pre-concentration and analysis. For these reasons, portable devices, such as biosensors [4,5], are required for on-site monitoring of heavy-metal ions. Enzymes are widely integrated in biosensors for sensitive determination of their substrates, inhibitors and/or competitors [6]. The entrapment of enzymes in electrosynthesised polymeric networks [7] is now commonly used for the development of biosensors [8]. Among several enzymes, the inhibition of glucose oxidase (GOx) has been extensively studied in the field of electrochemical biosensors for the detection of a wide set of inhibitors [9,10], including heavy-metal ions [11,12,13,14,15]. In addition, enzyme inhibition activities represent an attractive approach to producing a simple and rapid detection of the bioavailable fraction of heavy metal ions in water samples [16,17,18,19,20,21]. Owing to the complexity of these systems, the development of biosensors requires the studying of a set of parameters affecting their analytical responses. In the past, the most commonly-used approach to evaluating and optimising the influence of factors on the experimental responses was based on the univariate statistics. This technique precludes inclusion all the interactions among the variables studied in the experimental plan. The design of experiment (DOE) is useful to study all the interactions between the factors and to overcome the limits offered by a simple “one-at-a-time” approach [22,23,24,25]. It also helps the user to perform the optimisation of the parameters with a reduction of reagents consumption and number of experiments. In the literature, DOE has been little applied for the optimisation of biosensor performances [26,27,28,29,30]. In this work, the Pt/PPD/GOx biosensors, described by our previous works [31,32,33], were used in a flow injection setup and relevant responses were optimised. The effects of varying enzyme concentration (U·mL^−1^), the number of voltammetric cycles during the preparation of Pt/PPD/GOx biosensor and the flow rate (µL·min^−1^) on sensitivity (S, µA·mM^−1^) toward the new tested Bi^3+^ and Al^3+^ ions were investigated using response surface methodology (RSM) based on central composite design (CCD). We also report the performance of the biosensor for Ni^2+^ and Ag^+^ ions—already tested in our previous works—under the best selected conditions. Lineweaver-Burk and Dixon plots were reported to elucidate information about kinetic parameters and degree of inhibition on the studied biosensors toward previous studied ions.

## 2. Materials and Methods

### 2.1. Apparatus

Experiments were carried out with a computer controlled PalmSens potentiostat. All electrochemical data were collected and analysed by the PalmSens PSTrace software. The electrochemical cell was purchased from Dropsens and consisted of a plexiglass flow cell and a disposable screen ink-printed platinum electrode (SPPtE, DRP-150, Dropsens, Italy). They consisted in a platinum disk-shaped (12.6 mm^2^) working electrode, a paste of silver/silver chloride pseudo-reference and a platinum strip counter electrode, on a ceramic substrate (3.3 cm × 1.0 cm). All the electrochemical measurements were referred to the screen-printed silver pseudo-reference electrode. The screen printed electrodes were interfaced to the potentiostat by a cable connector for SPEs (DRP-CAC, Dropsens, Italy). A Gilson MiniPuls 3 peristaltic pump and a Rheodyne low pressure injector with a 200-µL sample loop completed the apparatus.

### 2.2. Chemicals

Glucose oxidase (GOx) from Aspergillus niger (Type VII, 248073 U/g) and D (+) glucose were purchased from Sigma-Aldrich. Bismuth standard solution (1000 ppm), Al(NO_3_)_3_, Hg(NO_3_)_2_, AgNO_3_, Ni(NO_3_)_2_ were purchased from Fluka. Acetate buffer (50 mM, pH = 5.2) was prepared from 50 mM acetic acid brought to pH 5.2 with NaOH. Glucose stock solutions were prepared in acetate buffer and were allowed to mutarotate overnight before use. 1 mM stock solution of metal ions were prepared in acetate buffer (50 mM, pH = 5.2) and diluted with the same buffer to give the required concentrations.

### 2.3. Preparation of the Pt/PPD/GOx biosensor

In order to prepare the biosensor, platinum screen-printed electrodes were chosen as the transducer element, as reported in our previous works [30,31,32,33]. Therefore, biosensors were grown on the surface working electrodes using a CV technique. Briefly, the surface of a platinum screen-printed electrode was washed with Milli-Q water. The electrodes were conditioned by cyclic voltammetry (CV) in 10 mM K_3_Fe(CN)_6_ solution between −0.3 V and +0.5 V until a steady state was reached. Thus, a drop of 50 µL of a solution containing variable concentration of GOx and 5 mmol/L of o-phenylenediamine were casted on the electrode surface, then a cyclic voltammetry between −0.07 V and +0.77 V was performed for the electrochemical grown of the polymer. The electrode was then rinsed with acetate buffer and mounted in the flow cell for the measurements. The Pt/PPD/GOx biosensors were stored in acetate buffer at 4 °C when not in use.

### 2.4. Electrochemical Estimation of Heavy Metal Ions

All the electrochemical measurements were carried out at room temperature in acetate buffer (50 mM, pH = 5.2, freshly prepared every week) at an applied potential of +0.47 V vs Ag/AgCl [31]. Next, 200 µL of glucose solution containing different amount of metal ions were injected in the flow injection apparatus and the inhibition of metal ions on glucose response was calculated with the following equation (Equation (1)):

Inhibition % = (I_0_ − I)/I_0_ × 100 (1)

where I and I_0_ represent the response of glucose on Pt/PPD/GOx biosensor with and without metal ions. All measurements were recorded in triplicate. The calibration curve was 1/i vs [I] (Dixon plot). The slopes were used to collect the sensitivity of the biosensor (S, µA·mM^−1^) towards metal ions in the optimisation procedure.

### 2.5. Experimental Design

Minitab 17 software (Minitab Inc., USA) was used for design, mathematical modelling, and optimisation. The independent factors (variables) considered in this work were flow rate, 0.3–1 mL min^−1^; number of cycles, 10–30; enzyme concentration, 50–800 U·mL^−1^ (X1, X2, and X3, respectively) (Table S1), whereas the sensitivities of the biosensor towards metal ions were the responses (dependent factors). oPD concentration (5 mmol·L^−1^ of o-phenylenediamine) during biosensor preparation and applied potential (0.47 V vs. Ag/AgCl) during response measurements were kept constant to avoid the influence of additional factors (more than three) and to have a simpler experimental design. Based on the CCD principle, the design consisted of ‘2k’ fractional factorial points plus ‘2k’ axial points and ‘1′ center point, where ‘k’ is the number of variables (in our case, k = 3). Thus, 20 experiments were conducted with 8 (23) fractional factorial points, 8 (2 × 4) axial points, and 6 replications of the central point to have a final estimation of the experimental error. A circumscribed design with the star and factorial points lying equidistant from the center was used. The range of the experimental domain was fixed onto the axial points. A second order polynomial (Equation (2)), consisting of linear, quadratic and first order interaction terms, was fitted to the measured individual response variables.

(2) $$y=\beta_{0}+\sum_{i=1}^{k}\beta_{i}x_{i}+\sum_{i=1}^{k}\beta_{ii}x_{i}^{2}+\;\sum_{i=1}^{k}\sum_{j\left(\neq i\right)=1}^{k}\beta_{ij}x_{i}x_{j}+\varepsilon$$

where *y* is one of the response variables (i.e., sensitivities), *x_i_* represent the dependent variables, *β*_0_; *β_i_*; *β_ii_*; *β_ij_* are the regression coefficients for intercept, linear, quadratic and interaction terms, respectively, *k* denotes the number of variables and *ε* represents the unexplained error. The regressions coefficients were estimated by the method of multiple-least square regression that finds the regression coefficients by minimising the sum of squares of the errors. The significance of the overall model, and of each regression coefficient was assessed by analysis of variance (ANOVA).

## 3. Results and Discussion

### 3.1. Glucose Responses and Inhibitive Detection of Heavy Metal Ions in A Fia Apparatus

**Figure 1:**
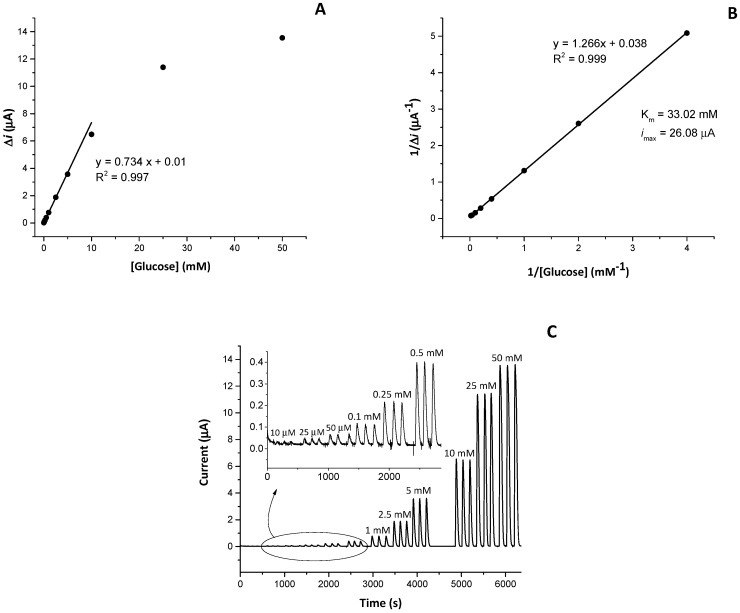
(**A**) Amperometric response of optimised Pt/PPD/GOx biosensor (50 U·mL^−1^, 30 cycles of CV) to glucose standard solution prepared in acetate buffer (0.05 M, pH = 5.2) and linear fit to the calibration curve (0.01–10 mM); (**B**) Lineaweaver-Burk plot; (**C**) FIA peaks recorded for triplicate injections of different concentrations of glucose (0.01–50 mM) at a flow rate of 0.3 mL·min^−1^.

The amperometric biosensors were prepared as reported elsewhere [33] by using different numbers of cycles during the electrosynthesis of the film and different enzyme concentrations. The FIA measurements were recorded in 50 mM acetate buffer (pH = 5.2) at the applied potential of 0.47 V and at different flow rates. The calibration curve to glucose at optimised conditions in the concentration range from 0.01 mM to 50 mM is reported in Figure 1A, whereas the FIA peaks recorded in the same concentration range were presented in Figure 1C. The linear range was from 10 µM to 10 mM, showing a sensitivity to glucose of 0.734 ± 0.010 mM·µA^−1^ (R^2^ = 0,997). Lineweaver-Burk plot (1/*i* vs 1/C) was used to determinate the apparent Michaelis-Menten constant, Km, as the glucose concentration at which the reaction rate is at half-maximum, and the maximum reaction rate achieved by the system in terms of current, *i*max (Figure 1B). The apparent K_m_ and the maximum rate *i*max were found to be 33.02 ± 0.08 mM (R^2^ = 0.999) glucose and 26.08 µA, respectively. The relationship Δ*i* vs. [glucose] curves after 10 mM and saturates at about 25 mM. The response of the biosensor is reproducible in the entire investigated range (RSD% =25 at 10 µM and RSD% = 0.21 at 50 mM), so that the sensor can be beneficial also at high glucose concentrations, which opens up opportunities for applications in food analysis.

**Figure 2:**
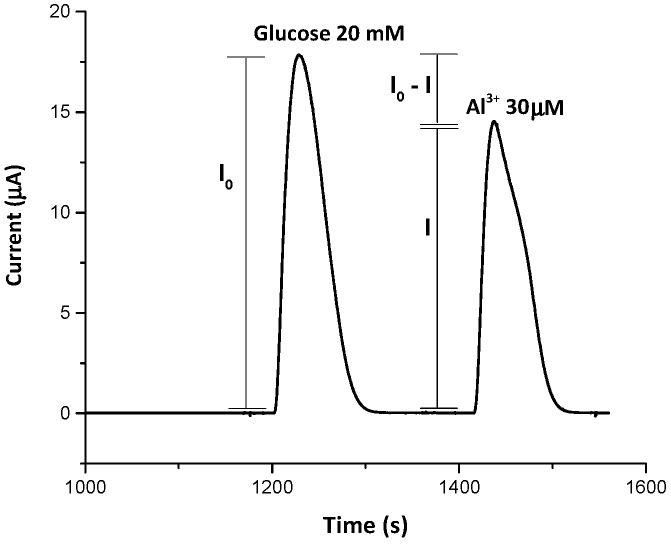
FIA peaks recorded for glucose (20 mM) and in presence of 30 µM of Al^3+^ ions prepared in acetate buffer (0.05 M, pH = 5.2). Experimental conditions as in Figure 1.

In order to show the degree of inhibition of the enzyme to heavy metal ions, we report a typical response of the biosensor to 30 µM of Al^3+^ ions (Figure 2).

### 3.2. Optimisation of the Performance of Biosensor Using DOE

Basically, the optimisation process involves three major steps: (1) performing the statistically designed experiments, (2) estimating the coefficients in a mathematical model, and (3) predicting the response and checking the appropriateness of the model. The electrochemical responses of a biosensor can be influenced by many experimental parameters that should be optimised in order to obtain better performances. The CCD was selected because it is a design that includes linear, quadratic and interaction terms and allows greater numbers of levels without performing experiments at every combination of factor levels [23].

Among the electrosynthesis parameters, the enzyme concentration and number of cycles were optimised. The amount of the enzyme and the number of cycles during the electrosynthesis were taken into account in order to understand if the (small) change in the film thickness can affect the polymer permselectivity and/or the amount of immobilised enzyme. The levels of these independent variables were selected starting from those used in previous works and extending their range in both sides (see Table S1). Flow rate also affects responses: on considering that low flow rate gave better responses [33], 1 mL·min^−1^, the flow rate used in the previous works, was retained as upper level whereas 0.3 mL·min^−1^ was selected as lower level. We chose to model the new tested metal ions (Bi^3+^ and Al^3+^) due to their potential to cause environmental damage. Table 1 shows the experimental design matrix along with measured sensitivities for Al^3+^ and Bi^3+^ for each set of independent variables.

The coefficients in a mathematical model (Equation (1)) were estimated by regression analyses and complete software output is reported in Supplementary Materials. The regression equations in uncoded units were (Equations (3) and (4)):

Y_1_ = −0.171 + 0.000214 X_1_ − 0.379 X_3_ + 0.0348 X_2_ + 0.000000 X_1_*X_1_ − 0.013 X_3_*X_3_ − 0.000191 X_2_*X_2_ + 0.000617 X_1_*X_3_ − 0.000057 X_1_*X_2_ + 0.0060 X_3_*X_2_ (3)

Y_2_ = 2.44 − 0.00464 X_1_ − 2.44 X_3_ + 0.0191 X_2_ + 0.000006 X_1_*X_1_ + 1.49 X_3_*X_3_ + 0.00117 X_2_*X_2_ + 0.00079 X_1_*X_3_ − 0.000131 X_1_*X_2_ − 0.0021 X_3_*X_2_ (4)

where Y_1_ is the sensitivity toward Al^3+^ and Y_2_ is the sensitivity toward Bi^3+^. ANOVA was also performed for each response, and the complete outputs are presented in Supplementary Materials. In a situation where a model fits the data, the mean square of the lack-of-fit error will be close to that of the pure error and the resulting ratio, the F-statistic, will be small. If the relevant P-value, i.e., the error probability, is large compared to the significance level (alpha value = 0.05), then the lack-of-fit is not significant, and the model adequately explains data in the region of experimentation. In the present case, the non-significant lack-of-fit for Al^3+^ (F = 2.39; P = 0.180 > 0.05) and Bi^3+^ (F = 3.38; P = 0.104 > 0.05) sensitivities suggest the adequacy of the model for both metal ions to explain data in the region of experimentation.

The Analysis of Variance tables (see Supplementary Materials) summarises the linear terms, the squared terms, and the interactions. The small p-values for the square or interaction terms suggest there is curvature in the response surface. According to the calculated p-values, the enzyme in the linear or square term of the model shows that this variable is statistically significant. Figure S1 shows the surface plots for metal ion sensitivities as function of enzyme concentration and number of cycles, where a curvature of responses is evident. In numerical optimisation, it is possible to choose the desired goal for each response. The overall desirability is an objective function ranging from 0 (if the optimal values are outside the selected ranges) to 1 (if the goal for each response is reached). All the sensitivities were maximised with the same weight and the composite desirability was equal to 1 at 50 U·mL^−1^ for enzyme concentration and 30 cycles during the synthesis of biosensor and 0.3 mL·min^−1^ for the flow rate during inhibition experiments.

To confirm the reliability of the model, additional laboratory experiments were conducted using the optimum conditions. The experimental results were in agreement with the predicted results (Table 2): it can be seen that the predicted confidence interval (PI) covered the values of sensitivity obtained from the experiments, confirming the validity of the model.

### 3.3. Analytical Performances of the Optimised Biosensor

**Figure 3:**
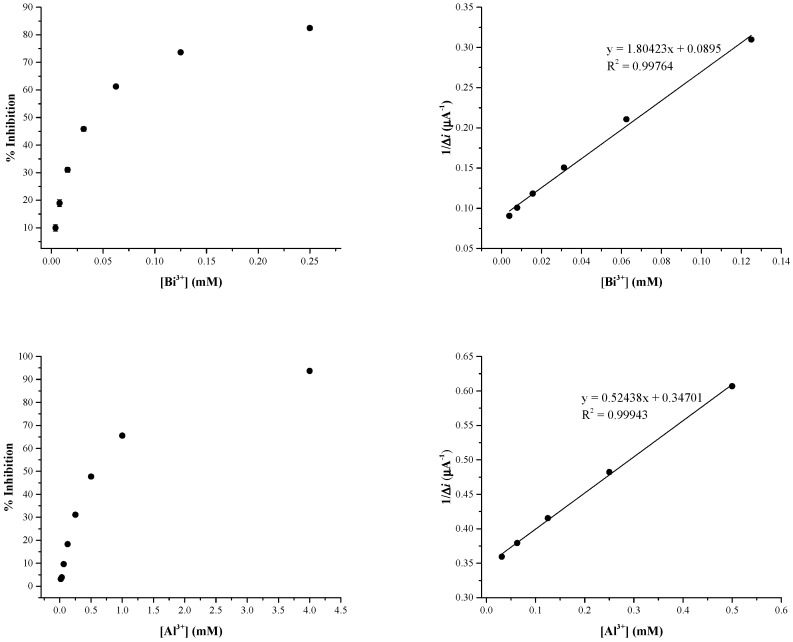
Inhibitive effects and Dixon plot for Pt/PPD/GOx biosensor after the injection of different concentration of Bi^3+^ and Al^3+^ ions in presence of 20 mM of glucose.

The inhibitive experiments were carried out at varying concentrations of Bi^3+^ (3.9 to 250 µM) and Al^3+^ (15.62 µM to 4 mM) in the presence of 20 mM of glucose dissolved in 50 mM acetate buffer (pH = 5.2). As a comparison with previous results, optimisation was also performed for Ag^+^ and Ni^2+^, obtaining the same optimal conditions (see below). Figure 3 shows the calibration graph constructed by plotting the inhibition percentage of the enzyme activity against the concentration of the new tested Bi^3+^ and Al^3+^ ions. 

Table 3 collects the detection limit (LOD, µM) which were evaluated according to recent advances considering the concentration of inhibitor which causes 10% of inhibition [16], instead of the frequently used 3x standard deviation (SD) of the blank. In the same table, the sensitivities of the biosensor obtained toward Bi^3+^ and Al^3+^ ions are reported.

**Figure 4:**
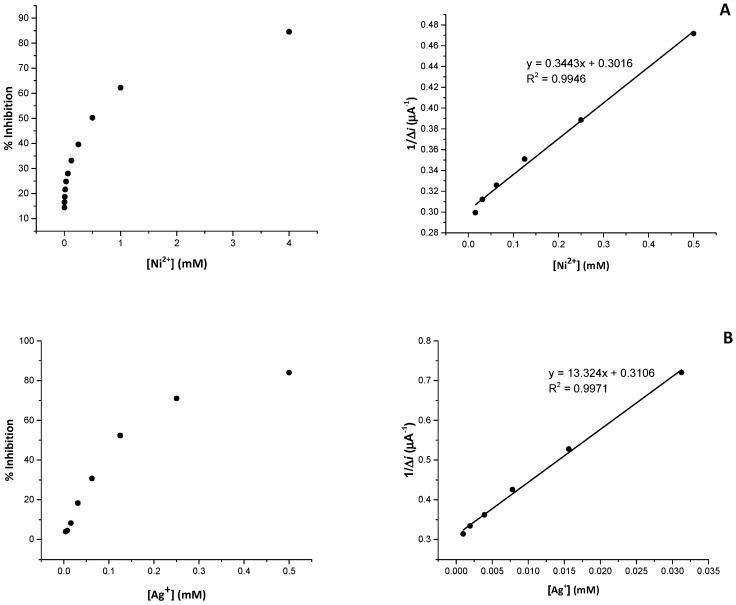
Inhibitive effects and Dixon plots for Pt/PPD/GOx biosensor after the injection of different concentration of Ni^2+^ (**A**) and Ag^+^ (**B**) in presence of 20 mM of glucose.

The performances of the optimal biosensor were also tested for Ni^2+^ and Ag^+^ ions, already investigated in our previous work [33]. While sensitivities were in well agreement with previous results, significant improvements were obtained for Ni^2+^ ions. In particular, we observed a single linear range for Ni^2+^ (Figure 4) in place of the two observed in the past (see reference [33]): possibly the lower amount of enzyme entrapped onto the electrode simplify the complex interaction mechanism between metal and enzyme. Also, the reusability of the biosensor was improved with respect to previous results (see below).

**Figure 5:**
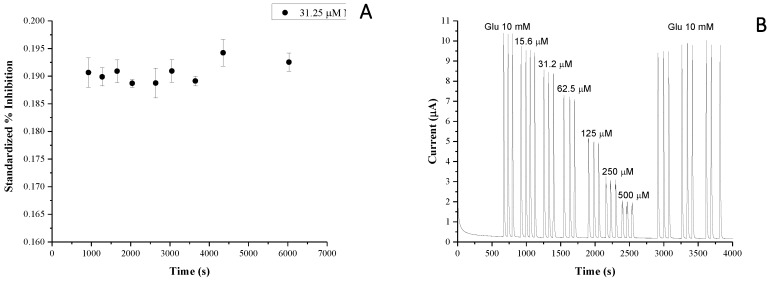

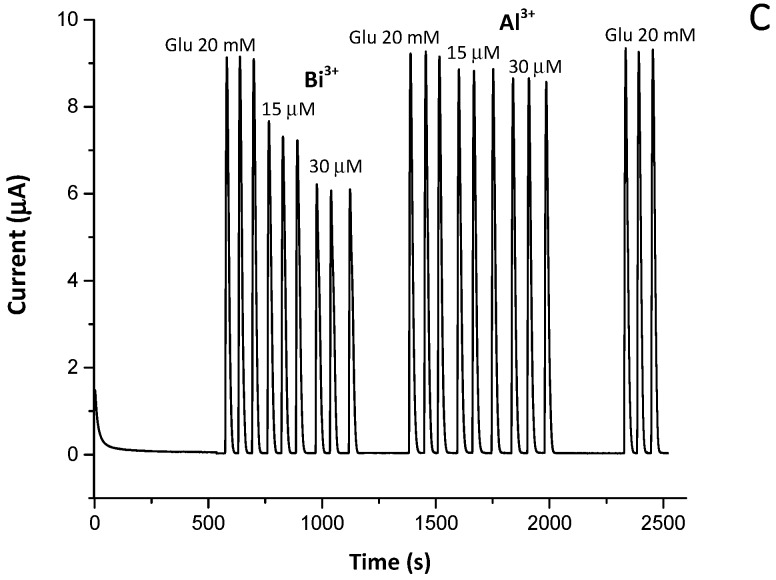
(**A**) Evaluation of working life of biosensor after subsequently injection of 31.25 µM of Ni^2+^. (**B**) Inhibition effect of Ag^+^ ions and recovery after washing with acetate buffer (50 mM, pH = 5.2). (**C**) Reusability of the biosensor after the injection of 15 µM and 31.25 µM of Bi^3+^ and Al^3+^ ions in presence of 20 mM of glucose and acetate buffer media.

Using the same system, the working and the storage stability were investigated; these were not evaluated in previous work. Figure 5A shows the working stability of the optimised PPD/GOx biosensor. Nine measurements over about 2 h were recorded with a low relative standard deviation (RSD) of 0.96%. The storage stability of the biosensor was studied by measuring current response at different times after conservation in acetate buffer at 4 °C in a refrigerator. The responses remained acceptable over more than 7 months, as the biosensor could keep 60% of its original current response with RSD of 0.72% for ten consecutive measurements. These results indicated that the Pt/PPD/GOx biosensor had good working and storage stability. 

The reversibility of enzyme activity is also important for the reusability of an inhibitive biosensor. The recovering of enzyme activity was tested by detecting the current response of the PPD/GOx biosensor towards glucose, before and after Ag^+^ detection [33]. Figure 5B shows the reusability of biosensors after the injection of different concentration of Ag^+^ ions (up to 0.5 mM) in the presence of 10 mM of glucose. It was possible to achieve 92% and 98% of recovery (slightly higher than the previous one [33]) of its initial current response after simply flushing the acetate buffer for 6 min and 11 min, respectively, without using any chelating agent, such as EDTA. Figure 5C shows the reusability of the biosensor after the injection of different concentrations of Bi^3+^ and Al^3+^ after the injection of glucose 20 mM. Also, in this case, we observed the recovery of the responses of the biosensor, confirming the possibility of reusing the sensor after a simple washing with the buffer media.

Even if the biosensors based on enzymes as biological receptors were extensively studied in the literature, these devices suffer from poor selectivity towards the inhibitors of the enzymatic activity. For instance, the optimised biosensor responds towards Zn^2+^, Cd^2+^, Cr^3+^ and Co^2+^ ions. So, these devices are a portable alternative for the in-situ monitoring of the contaminants, without requiring any sample preparation and transportation; furthermore, the biosensors could be successfully used in screening analysis for the total contamination of metal ions in water samples. The analytical application of the biosensors based on enzyme inhibition is still limited, since these devices were affected by various toxic compounds in the same sample. As an example, the inhibitive activity on different enzymes against herbicides is reported in the literature [34]. In our case, the selectivity of the biosensor was evaluated in the presence of atrazine and 4-(2,4-dichlorophenoxy) butyric acid (2,4-DB). Any inhibition effect was visible after the injection of 0.8 µM of atrazine and 39.6 µM of 2,4-DB (Figure S3), confirming the application of the biosensor for the selective detection of metal ions.

## 4. Conclusions

In this work, the performance of an amperometric biosensor was successfully maximised using an experimental design. The inhibitive behaviour of our previously studied biosensor was modelled by response surface methodology, and selected experimental parameters were optimised by a CCD design. The optimal setup involved a GOx concentration of 50 U·mL^−1^ and 30 cycles during the electrosynthesis of the biosensor, whereas 0.3 mL·min^−1^ was the optimal flow rate of measurements. The model was validated and employed successfully to also detect Al^3+^ and Bi^3+^, two metal ions whose inhibition capability is here presented for the first time. The inhibitive biosensor was demonstrated to have similar sensitivities compared to the previous biosensor, but improved reproducibility, stability and reversibility. In this configuration, it can be effectively used as gross biosensor to detect heavy metal pollution. Furthermore, thanks to its stability and reversibility, the sensor can be used as amperometric detector in ion chromatographic system to overcome the lack of selectivity.

## Figures and Tables

**Table 1:** Experimental plan and sensitivity in the range of concentration between 15.62 µM and 31.25 µM reported for Al^3+^ and Bi^3+^.

Experiment			Factors			Responses (Sensitivity, µA·mM^−1^)	
RdesStdOrder	RunOrder	PtType	Enzyme Concentration (U/mL)	Flow Rate (mL/min)	Number of Cycles during CV	Al^3+^	Bi^3+^
3	1	1	202	0.86	14	0.068	1.059
12	2	−1	425	1	20	0.035	0.657
14	3	−1	425	0.65	30	0.026	0.509
20	4	0	425	0.65	20	0.060	0.438
19	5	0	425	0.65	20	0.025	0.454
11	6	−1	425	0.3	20	0.077	0.599
5	7	1	202	0.44	26	0.417	1.675
1	8	1	202	0.44	14	0.074	1.222
13	9	−1	425	0.65	10	0.051	0.616
16	10	0	425	0.65	20	0.225	0.443
7	11	1	202	0.86	26	0.323	1.553
15	12	0	425	0.65	20	0.124	0.541
10	13	−1	800	0.65	20	0.033	0.838
2	14	1	648	0.44	14	0.078	0.697
17	15	0	425	0.65	20	0.094	0.738
9	16	−1	50	0.65	20	0.167	1.642
6	17	1	648	0.44	26	0.001	0.506
4	18	1	648	0.86	14	0.069	0.532
8	19	1	648	0.86	26	0.139	0.479
18	20	0	425	0.65	20	0.118	0.579

**Table 2:** Optimal and laboratory experiment validation.

	Experimental Value (µA/mM)	Fit (µA/mM)	95% PI (From Design)
S Bi^3+^	1.804	2.684	(1.759; 3.608)
S Al^3+^	0.524	0.574	(0.095; 1.054)

**Table 3:** Analytical data collected from Dixon plots obtained for Bi^3+^, Al^3+^ ion in presence of 20 mM of glucose.

Metal Ion	LOD (µM)	Upper limit of Linearity (µM)	Sensitivity (µA mM^−1^)
Bi^3+^	3.9	125	1.80 ± 0.12
Al^3+^	16	500	0.52 ± 0.02

## Supplementary Materials

The following are available online at http://www.mdpi.com/2079-6374/9/1/26/s1, Table S1: Variables and levels considered for the design of experiment (DOE), Figure S1: Response Surface for Al^3+^ (A), and Bi^3+^ (B): the sensitivities were improved when low concentrations of enzyme and a high number of cycles were employed during the synthesis of biosensor, Figure S2: Numerical optimisation performed by the software, Figure S3: Electrochemical responses of PPD/GOx biosensor after the injection of 0.8 µM of atrazine and 36.8 µM of 2,4-DB. Baseline: Glucose 1 mM.
