# Supplementary material for: Response Surface Methodology for the Optimisation of Electrochemical Biosensors for Heavy Metals Detection
Source: Biosensors (Basel). 2019 Feb 13;9(1):26. doi: 10.3390/bios9010026 (PMC6468913; doi:10.3390/bios9010026)
Supplement: Supplementary file 1 [file biosensors-09-00026-s001.pdf]

Supplementary Materials

Article

Response Surface Methodology for the Optimisation of Electrochemical Biosensors for Heavy Metals Detection

Giuseppe Egidio De Benedetto [1], Sabrina Di Masi [2,*], Antonio Pennetta [1] and Cosimino Malitesta [2]

[1] Dipartimento di Beni Culturali, Università del Salento, Via D. Birago 64, 73100 Lecce, Italy; giuseppe.debenedetto@unisalento.it (G.E.D.B.); antonio.pennetta@unisalento.it (A.P.)
[2] Dipartimento di Scienze e Tecnologie Biologiche ed Ambientali, Via per Monteroni 1, 73100 Lecce, Italy; cosimino.malitesta@unisalento.it
* Correspondence: sabrina.dimasi@unisalento.it; Tel.: +39-0832-29-7145

Received: 11 January 2019; Accepted: 09 February 2019; Published: 13 February 2019

Contents

1. Table S1
2. Analysis of Variance of the model for Al^{3+} and Bi^{3+}
3. Optimisation of responses
4. Selectivity of the biosensor

1. Table S1

Table S1. Variables and levels considered for the design of experiment (DOE).

Name (Factor)	Units	Low	High
**Enzyme concentration (X1)**	U/mL	50	800
**Number of CV cycles (X2)**	-	10	30
**Flow rate (X3)**	mL/min	0.3	1.0

2. Analysis of Variance of the Model for Al^{3+} and Bi^{3+}

The analysis of variance with the calculated F and p values were obtained for all the tested metal ions.

Response Surface Regression: Sensitivity towards Al^{3+} ions versus [Enzyme] (U/mL); Flow rate (mL/min); Number of cycles

Analysis of Variance.

Source	DF	Adj SS	Adj MS	F-Value	P-Value
Model	9	0.128467	0.014274	1.79	0.188
Linear	3	0.071472	0.023824	3.00	0.082
[Enzyme] (U/mL)	1	0.049279	0.049279	6.20	0.032
Flow rate (mL/min)	1	0.000127	0.000127	0.02	0.902
Number of cycles	1	0.022066	0.022066	2.77	0.127
Square	3	0.004253	0.001418	0.18	0.909
[Enzyme] (U/mL)*[Enzyme] (U/mL)	1	0.003239	0.003239	0.41	0.538

Flow rate (mL/min)*Flow rate (mL/min)	1	0.000005	0.000005	0.00	0.981
Number of cycles*Number of cycles	1	0.000657	0.000657	0.08	0.780
2-Way Interaction	3	0.052743	0.017581	2.21	0.150
[Enzyme] (U/mL)*Flow rate (mL/min)	1	0.006555	0.006555	0.82	0.385
[Enzyme] (U/mL)*Number of cycles	1	0.045753	0.045753	5.75	0.037
Flow rate (mL/min)*Number of cycles	1	0.000435	0.000435	0.05	0.820
Error	10	0.079532	0.007953		
Lack-of-Fit	5	0.056098	0.011220	2.39	0.180
Pure Error	5	0.023433	0.004687		
Total	19	0.207999			

Model Summary.

S	R-sq	R-sq(adj)	R-sq(pred)
0.0891806	61.76%	27.35%	0.00%

Fits and Diagnostics for Unusual Observations.

Obs	S_Al3+	Fit	Resid	Std	Resid
3	0.0260	0.1535	-0.1275	-2.28	R
7	0.4170	0.3128	0.1042	2.03	R

R: Large residual.

Residual Plots for S_Al3+

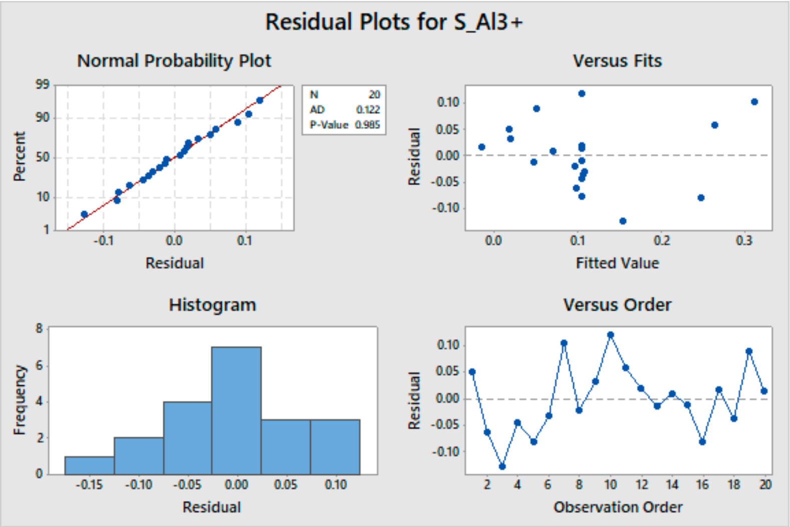

Response Surface Regression: Sensitivity towards Bi3+ versus [Enzyme] (U/mL); Flow rate (mL/min); Number of cycles.

Analysis of Variance.

Source	DF	Adj SS	Adj MS	F-Value	P-Value
Model	9	2.93908	0.32656	11.04	0.000
Linear	3	1.61191	0.53730	18.16	0.000
[Enzyme] (U/mL)	1	1.58134	1.58134	53.44	0.000
Flow rate (mL/min)	1	0.01054	0.01054	0.36	0.564
Number of cycles	1	0.02003	0.02003	0.68	0.430
Square	3	1.14477	0.38159	12.90	0.001
[Enzyme] (U/mL)*[Enzyme] (U/mL)	1	1.12542	1.12542	38.03	0.000
Flow rate (mL/min)*Flow rate (mL/min)	1	0.05734	0.05734	1.94	0.194
Number of cycles*Number of cycles	1	0.02296	0.02296	0.78	0.399
2-Way Interaction	3	0.18240	0.06080	2.05	0.170
[Enzyme] (U/mL)*Flow rate (mL/min)	1	0.00108	0.00108	0.04	0.852

[Enzyme] (U/mL)*Number of cycles	1	0.17731	0.17731	5.99	0.034
Flow rate (mL/min)*Number of cycles	1	0.00401	0.00401	0.14	0.721
Error	10	0.29591	0.02959		
Lack-of-Fit	5	0.22835	0.04567	3.38	0.104
Pure Error	5	0.06757	0.01351		
Total	19	3.23499			

Model Summary.

S	R-sq	R-sq(adj)	R-sq(pred)
0.172022	90.85%	82.62%	43.41%

Fits and Diagnostics for Unusual Observations.

Obs	S_Bi3+	Fit	Resid	Std	Resid
7	1.675	1.455	0.220	−2.23	R
16	1.642	1.890	−0.248	2.30	R

R: Large residual.

Residual Plots for S_Bi3+

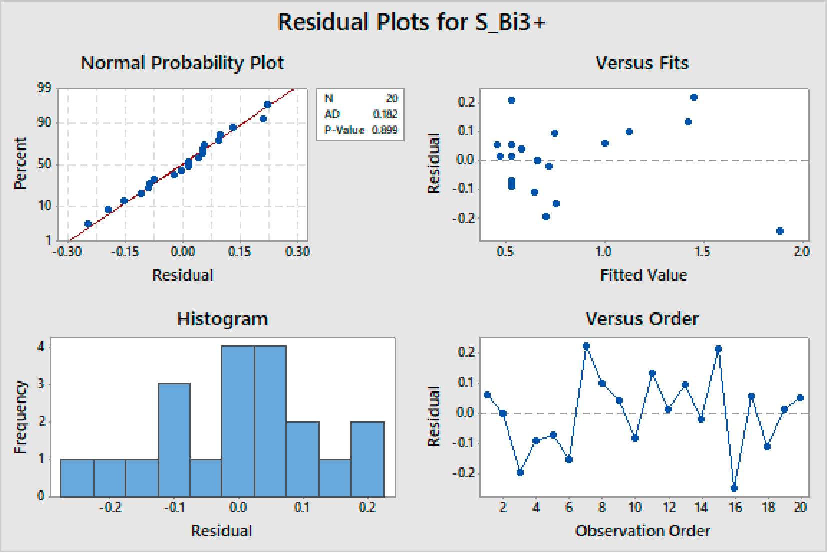

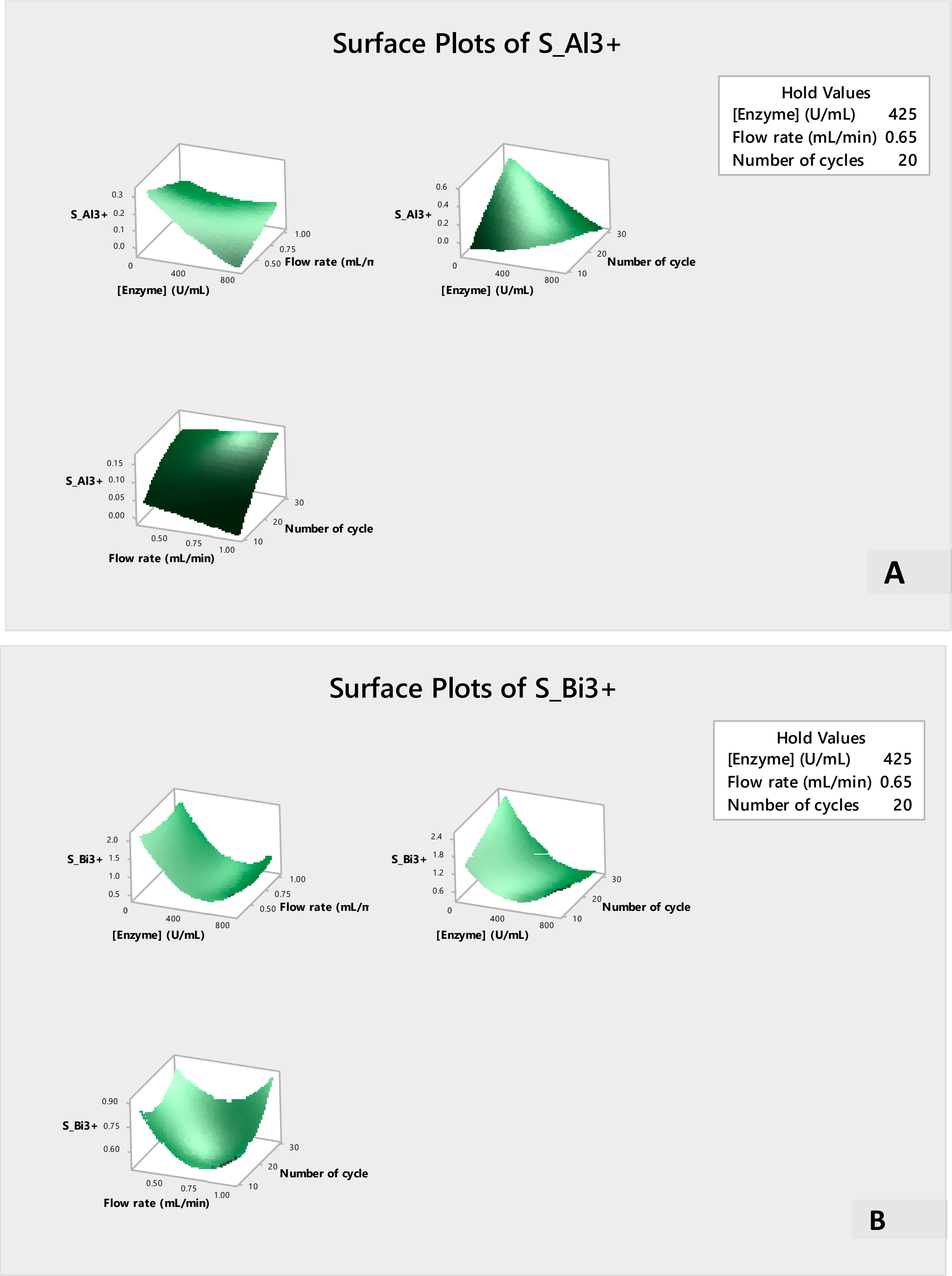

Figure S1. Response Surface for Al^{3+} (A), and Bi^{3+} (B): the sensitivities were improved when low concentrations of enzyme and a high number of cycles were employed during the synthesis of biosensor.

3. Optimisation of the responses

Response Optimization: S_Bi³⁺; S_Al³⁺.

Response	Goal	Lower	Target	Upper	Weight	Importance
S_Bi^{3+}	Maximum	0.438	1.675		1	1
S_Al^{3+}	Maximum	0.001	0.417		1	1

Optimization Plot

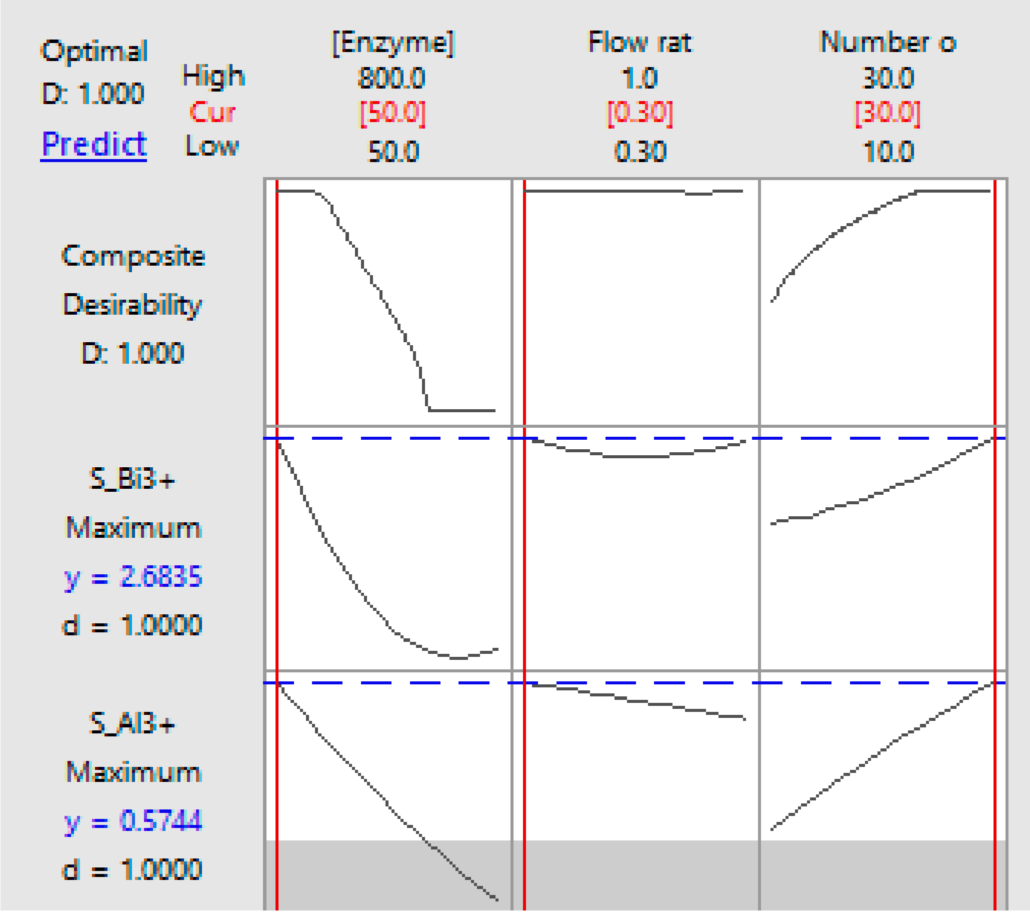

Figure S2. Numerical optimisation performed by the software.

Solution.

Solution	[Enzyme](U/mL)	Flow rate (mL/min)	Number of cycles	S_Bi3+	S_Al3+	Composite Desirability
1	50	0.3	30	2.68352	0.574429	1

4. Selectivity of biosensor

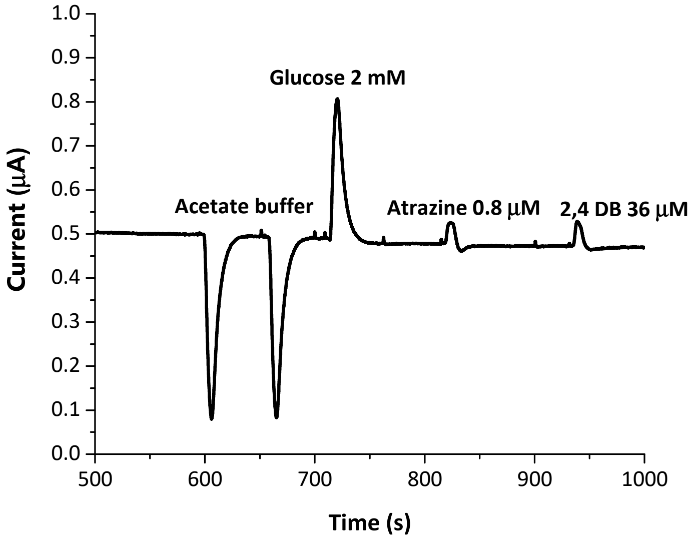

Figure S3. Electrochemical responses of PPD/GOx biosensor after the injection of 0.8 µM of atrazine and 36.8 µM of 2,4-DB. Baseline: Glucose 1 mM.
